# Role-reversed polyandry is associated with faster fast-Z in shorebirds

**DOI:** 10.1098/rspb.2024.0397

**Published:** 2024-06-12

**Authors:** Kees Wanders, Guangji Chen, Shaohong Feng, Tamás Székely, Arraxi O. Urrutia

**Affiliations:** ^1^ Department of Life Sciences, Milner Centre for Evolution, University of Bath, Bath, UK; ^2^ Department of Evolutionary Zoology and Human Biology, HUN-REN-DE Reproductive strategies Research Group, University of Debrecen, Debrecen, Hungary; ^3^ Natural History Museum of Denmark, University of Copenhagen, Copenhagen, Denmark; ^4^ Center for Evolutionary & Organismal Biology, Liangzhu Laboratory, Department of General Surgery, Sir Run Run Shaw Hospital, Zhejiang University School of Medicine, Hangzhou, People’s Republic of China; ^5^ BGI Research, Wuhan, People’s Republic of China; ^6^ College of Life Sciences, University of Chinese Academy of Sciences, Beijing, People’s Republic of China; ^7^ Debrecen Biodiversity Centre, University of Debrecen, Debrecen, Hungary; ^8^ Instituto de Ecologia, UNAM, Mexico City, Mexico

**Keywords:** fast-Z, genetic drift, Z chromosome, polyandry, sexual selection, shorebirds

## Abstract

In birds, males are homogametic and carry two copies of the Z chromosome (‘ZZ’), while females are heterogametic and exhibit a ‘ZW’ genotype. The Z chromosome evolves at a faster rate than similarly sized autosomes, a phenomenon termed ‘fast-Z evolution’. This is thought to be caused by two independent processes—greater Z chromosome genetic drift owing to a reduced effective population size, and stronger Z chromosome positive selection owing to the exposure of partially recessive alleles to selection. Here, we investigate the relative contributions of these processes by considering the effect of role-reversed polyandry on fast-Z in shorebirds, a paraphyletic group of wading birds that exhibit unusually diverse mating systems. We find stronger fast-Z effects under role-reversed polyandry, which is consistent with particularly strong selection on polyandrous females driving the fixation of recessive beneficial alleles. This result contrasts with previous research in birds, which has tended to implicate a primary role of genetic drift in driving fast-Z variation. We suggest that this discrepancy can be interpreted in two ways—stronger sexual selection acting on polyandrous females overwhelms an otherwise central role of genetic drift, and/or sexual antagonism is also contributing significantly to fast-Z and is exacerbated in sexually dimorphic species.

## 1. Background

Z chromosomes are part of the heterogametic (ZW) sex-determination system—females have dissimilar sex chromosomes, being ZW, while males are homogametic (ZZ). All bird species share the same ZW determination system that originated around 140 Ma [1]. Moths and butterflies, several snake lineages and a handful of fish species also have a ZW sex-determination system [2]. In contrast to the W chromosome, which is highly degenerate with a high content of repetitive sequences and few protein-coding genes, Z chromosomes are large in size, rich in protein-coding regions and more closely resemble autosomal chromosomes. However, despite this similarity, Z chromosomes exhibit a higher ratio of non-synonymous substitutions (changes in the coding sequence that result in a change to the amino acid encoded) to synonymous substitutions (changes that do not affect the amino acid sequence), relative to the autosomes. This is indicative of a higher rate of evolutionary change on the Z chromosome—a pattern termed as ‘fast-Z’ evolution [3–5].

Evidence for fast-Z evolution is strong in birds, and it has been detected in a number of Galloanserae (fowl) species [6–8], as well as in tinamous [9] and many Passeriformes [10–13]. To date, the only avian clade where fast-Z effects appear to be missing is the ratites, which may be unsurprising, given that the ratite W chromosome contains large ‘pseudo-autosomal regions’, where normal function and recombination with the Z chromosome are maintained [9]. Fast-Z has also been detected in two snake lineages [14] as well as four lepidopterans [15–17], although it was not detected in another two lepidopterans [18]. A similar phenomenon is observed in species with an XY sex-determination system, whereby the X chromosome experiences higher rates of evolution compared with autosomal chromosomes across a wide range of animal taxa [19].

Two major theories explaining fast-Z and fast-X effects have been put forward. The first suggests that the heterogametic sex (the female in a ZW sex-determination system) is exposed to stronger selection on partially recessive alleles because the degeneration of the W chromosome means that only one copy of most Z chromosome genes is carried [20]. Under this theory, the high rate of non-synonymous substitutions on the Z chromosome reflects strong positive selection on new beneficial alleles, which are less efficiently fixed on the autosomes due to masking of their recessive effects. Alternatively, it has been suggested that the lower effective population size of the Z chromosome (three-quarters that of the autosomes, under random mating conditions and assuming similar recombination rates) reduces the efficiency of purifying selection on the Z chromosome [21,22]. According to this theory, the high rate of non-synonymous substitutions on the Z chromosome instead reflects weak purifying selection on harmful alleles, which are fixed more often on the Z chromosome owing to genetic drift.

The study of polymorphisms (genome sequence variation within populations) can help to disentangle which of the two processes potentially underlying fast-Z is most influential in birds. Unlike fixed substitutions, which reflect differences between species that include a mixture of adaptive and harmful alleles, non-synonymous polymorphisms are typically harmful alleles that remain in a population owing to genetic drift [23]. If fast-Z is primarily caused by particularly efficient selection on partially recessive alleles, we would therefore predict fewer non-synonymous polymorphisms on the Z chromosome compared with autosomes, as they should be more efficiently purged owing to their exposure to selection in females. By contrast, if fast-Z is primarily caused by weak purifying selection owing to a low Z chromosome effective population size, we would predict an excess of non-synonymous polymorphisms on the Z chromosome.

Research to date has found that the Z chromosome in birds typically carries an excess of non-synonymous polymorphisms relative to the autosomes, implicating genetic drift as the mechanism driving fast-Z in birds [6,10,11,13] (although see [12] for more mixed results across three species of sparrow). Direction-of-selection analysis directly compares the frequency of polymorphisms with the frequency of substitutions to determine whether substitutions are fixed by the action of drift or positive selection [23]. In this test, it is expected that stronger positive selection would result in a higher rate of non-synonymous substitutions (corrected by synonymous substitutions) compared with the number of non-synonymous polymorphisms (corrected by the number of synonymous polymorphisms). The opposite would be expected if the action of drift is driving the accumulation of non-synonymous substitutions. Studies taking this approach in Lepidoptera have found an excess of non-synonymous substitutions relative to non-synonymous polymorphisms on the Z chromosome, relative to the autosomes, suggestive of more efficient positive selection on partially recessive alleles contributing to fast-Z [15–18]. Similarly, a study in Galloanserae birds using a direction-of-selection approach applied to gene expression (as opposed to coding sequence evolution) found that a higher proportion of gene expression evolution is explained by positive selection on the Z chromosome, relative to the autosomes [24]. Thus, results from coding sequence and gene expression analyses indicate that positive selection also contributes to ‘fast-Z’ effects. This is consistent with studies examining ‘fast-X’ effects, which tend to support a significant role of positive selection in driving rapid X-chromosome evolution [19,25]. Altogether, the research comparing within-population diversity with between-population divergence suggests that the relative contributions of genetic drift and positive selection in driving fast-Z or fast-X evolution vary according to the lineage and the form of evolution under investigation [26].

The study of mating systems provides a conceptually different approach to disentangling the mechanisms underlying fast-Z in birds, by considering how sex differences in the variance of reproductive success affect the Z chromosome’s effective population size. Under polygyny (polygamy in males), the effective population size of the Z chromosome is particularly low, as males carry most of the Z chromosome copies and there are fewer breeding males than females. This means that under extreme polygyny where few males monopolize many females, *N*_*eZ*_ ~ ½*N*_*eA*_, while under monogamy, *N*_*eZ*_ ~ ¾*N*_*eA*_ [21,22]. By contrast, under role-reversed polyandry (an unusual system for birds in which few females monopolize many males [27]), the effective population size of the Z chromosome is expected to be more similar to that of the autosomes (*N*_*eZ*_ ~ *N*_*eA*_) [22]. Note that the precise relationship between *N*_*eZ*_ and *N*_*eA*_ depends also on the recombination rates for these genomic regions [28]. However, assuming such recombination rate differences remain consistent across mating systems, the effective population size of the Z chromosome, relative to the autosomes, will be lowest under polygyny and highest under polyandry.

In line with these theoretical predictions, polygynous shorebird, grouse and flycatcher species have been found to exhibit particularly low *N*_*eZ*_/*N*_*eA*_ ratios compared with expectations under monogamy [10,29–31], while polyandrous jacana species exhibit the highest *N*_*eZ*_/*N*_*eA*_ ratios recovered to date [32]. If fast-Z is driven by weak purifying selection owing to the genetic drift on the Z chromosome, we may therefore predict that polygynous species should show a particularly strong fast-Z effect, and polyandrous species should show a particularly weak fast-Z effect. Consistent with genetic drift driving fast-Z in birds, polygynous Galliformes do indeed show a particularly high ratio of non-synonymous to synonymous substitutions on the Z chromosome, relative to monogamous species [6]. However, the prediction that polyandrous species should show a particularly weak fast-Z effect (if genetic drift drives fast-Z) remains untested.

Here, we address this gap in the literature by investigating how fast-Z evolution is impacted by role-reversed polyandry in shorebirds, a paraphyletic group of wading birds within the Charadriiformes order, which are notable for exhibiting a tremendous variety of mating systems [33,34]. To achieve these aims, we first test whether shorebird clades have been evolving under role-reversed polyandry for sufficiently long to expect a genomic signature of this mating system. Next, we align orthologues from 23 published shorebird genomes and compare the substitution rates for genes on the Z chromosome with those on the autosomes. If genetic drift and weak purifying selection are the primary forces underlying fast-Z, we expect role-reversed polyandrous species to show a weaker fast-Z effect than non-reversed species, as the high variance in female reproductive success erodes the difference in effective population size between the Z chromosome and the autosomes [22]. By contrast, if positive selection on partially recessive alleles in females is driving the fast-Z effect, we may predict even stronger fast-Z in role-reversed polyandrous species than monogamous species. This is because polyandrous females are under particularly strong sexual selection, and so the exposure of partially recessive alleles to selection in females may be particularly influential for these species [35,36].

Finally, we briefly investigate the relationship between role-reversed polyandry and the synonymous substitution rate on the autosomes and Z chromosome to test whether polyandry accelerates the rate of neutral evolution owing to higher germline mutation rates. This has been hypothesized to occur via numerous mechanisms, including a mutational effect of sperm competition, selection for the mutation of rare beneficial alleles or trade-offs between DNA repair mechanisms and sexual selection [37–40]. A mutational effect of sperm competition would also disproportionately affect the Z chromosome synonymous substitution rate and may also contribute to fast-Z, since the Z chromosome is most often found in males [41,42].

## 2. Methods

### (a) Mating system characterization

The impact of role-reversed polyandry on the female variance in reproductive success (relative to male variance in reproductive success) depends on the extent of polygamy among females and males. These characteristics vary widely even among polyandrous bird species, e.g. estimates of the proportion of breeding females engaging in polygamy vary from 50 to 100% among polyandrous jacana species, while the proportion of males engaging in polygamy varies from 0 to 40% [43]. Here, we apply a particularly strict definition to ‘role-reversed polyandry’ to include only species where >20% of breeding females are polygamous within a season, where <1% of breeding males are polygamous in a season, and where the majority of parental care is performed by the male. Species were assigned using data from Székely *et al*. [44], and assignments were based on scores of the social mating system (based on pair bonds and courtship), which in shorebirds aligns closely with the genetic mating system (which is based also on extra-pair mating) [45,46]. The strict definition used to determine ‘role-reversed polyandry’ strengthens the prediction of greater female than male variance in reproductive success [47], but it excludes some species that are often considered to exhibit role-reversed polyandry (e.g. Wilson’s phalarope *Phalaropus tricolor* and the red-necked phalarope *Phalaropus lobatus*, both of which exhibit relatively low rates of female polygamy despite male-only parental care). The wattled jacana *Jacana jacana* is the best-studied role-reversed polyandrous species included in this study, and it is characterized by ~1.7 breeding males per female, with ~60% of females mating polygamously within a season and 0% of males mating polygamously [48].

The presence of male–male competition and sperm competition in the wattled jacana, or in other role-reversed polyandrous species, has the potential to elevate the variance in reproductive success in males—some males may gain reproductive success through paternity of nests incubated by other males breeding in the same female’s territory. Such an effect could hypothetically lower the male effective population size and result in unclear predictions as to whether the female or male effective population size is greater, even in the absence of male polygamy. However, the few studies that have quantified the rate of extra-pair paternity (EPP) in role-reversed polyandrous shorebirds have found fairly low rates of EPP: 7.5% of chicks are from extra-pair males in the wattled jacana [49], 3.6% in the red phalarope *Phalaropus fulicarius* [50], 4.6% in the Eurasian dotterel *Charadrius morinellus* [51] and 8.6% in the spotted sandpiper *Actitis macularius* [52]. These EPP rates are within the range found among monogamous shorebird populations and are far lower than the rates seen under polygynandry (polygamy in both sexes) [46,53].

Observational studies in the wattled jacana have further suggested that EPP in this species occurs randomly with respect to male phenotype and that females show no mating preference towards individual partners [48]. Under such conditions, with EPP distributed randomly across males, sperm competition and male–male competition have little impact on the variance in male reproductive success. Therefore, we believe that the impact of male–male competition and sperm competition is unlikely to remove the general expectation of greater female than male variance in reproductive success under our strict definition of role-reversed polyandry. Also consistent with this expectation, estimates of the *N*_*eZ*_/*N*_*eA*_ ratio based on polymorphism data exceed 1 for the two jacana species where this has been quantified (the African jacana *Actophilornis africanus* and the Madagascar jacana *Actophilornis albinucha*) [32].

The set of 23 shorebird genomes analysed in this study includes 4 role-reversed polyandrous species (>20% female polygamy and <1% male polygamy), 12 monogamous species (<1% polygamy in either sex), 5 species exhibiting various other mating systems (‘lekking polygyny’: >20% male and female polygamy, with male lekking behaviour; ‘polygynandry’: >20% male and female polygamy, without lekking behaviour; and ‘weak polyandry’: >5% female polygamy, with >1% male polygamy), as well as two species with unknown mating systems. Role-reversed polyandry is expected to result in the greatest Z chromosome effective population size (relative to autosomal effective population size) when compared with all other mating systems [22]. However, the strength of this effect depends on the mating systems of the comparison species, so for clarity, we provide two comparisons throughout our analyses: (i) role-reversed polyandry versus all other known mating systems and (ii) role-reversed polyandry versus monogamy.

### (b) Genomes, orthology and phylogeny

The 23 published shorebird genomes, 11 seabird genomes (non-shorebird charadriiforms) and 2 more distant genomes (chicken *Gallus gallus* and zebra finch *Taeniopygia guttata*) were obtained from the B10k project for this analysis [54]. Protein coding gene sequences were obtained from the genomes based on annotations, and orthologues were identified by blasting all 16 027 annotated chicken genes against each of the genomes and then blasting all annotated genes from each individual genome against the chicken genome. Only those that constituted reciprocal best hits were considered further in the study, and 4106 complete orthologues were identified across all 36 species [55].

**Figure 1.**
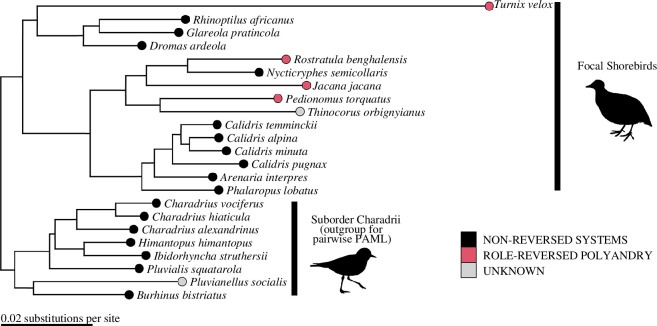
Phylogeny of genome-sequenced shorebirds with mating system information (role-reversed polyandry shown in pink versus non-reversed mating systems in black, and unknown mating systems in grey). The outgroup and focal shorebirds used for the pairwise substitution rate analyses are labelled. Edge lengths represent mean expected substitutions per site in the 4106 orthologous genes used to create the tree (scale bar, bottom left). The exceptionally high substitution rate in *Turnix velox* probably reflects the short generation time in this species, which reaches maturation just three months after hatching [58].

Phylogenetic relationships between species were inferred using two different methods based on the orthologue genes: a concatenation-based approach via RAXML (v. 8.2.4) using either full orthologue sequences or only fourfold degenerate sites [56], and a coalescence-based approach via ASTRAL-III (v. 5.14.2) using full orthologue sequences [57]. All methods produced identical topologies with regards to the shorebird phylogeny (figure 1). However, there was some inconsistency in the placement of the black skimmer *Rynchops niger,* which was placed either outside the clade containing gulls and terns, or as a closer relative of terns than gulls. The branch lengths estimated using RAXML v. 8.2.4, under a GTR + GAMMA substitution model in substitutions per site on the concatenated orthologue alignment sequences, were retained for subsequent analysis steps.

### (c) Substitution rate analysis overview

There were 5239 orthologues >100 bp in length found in all 23 shorebird genomes, and these were used to analyse the strength and presence of fast-Z. PRANK and Pal2nal were used to transform the gene alignments into ungapped codon alignments [59,60], and two complementary PAML codeml models were used to estimate the substitution rates [61]. The filtering of data and subsequent statistical analyses were all completed in R v. 4.2.2 [62].

Firstly, a free-ratios model was applied using the RAXML phylogeny based on whole-orthologue sequences, which provided estimates of the number of non-synonymous (*D*_*N*_) and synonymous (*D*_*S*_) substitutions in each branch visible in figure 1 as well as quantifying the number of sites where non-synonymous and synonymous substitutions were possible. These results were extracted for all terminal branches (tree tips) using a custom R script, providing estimates of the number of non-synonymous and synonymous substitutions for each species’ recent evolutionary history (note this included both the ‘focal’ and ‘outgroup’ species labelled in figure 1). This approach provides phylogenetically independent estimates of the substitution rate for the terminal branches leading to each shorebird species, but this is potentially confounded by the differences in terminal branch lengths among species. Furthermore, because orthologues are concatenated in order to detect enough substitutions for an accurate *D*_*N*_/*D*_*S*_ ratio, the calculated substitution rate is particularly reflective of long and fast-evolving genes. The results generated from the free-ratios model are also dependent on an accurate tree topology.

To account for the potential confounds in substitution rate estimates obtained from the free-ratios PAML model, a pairwise PAML model was also applied to the orthologues. For this approach, the eight shorebirds from the Charadrii suborder were used as outgroup species. Substitutions between the outgroup species and the 14 shorebirds from the Scolopaci and Lari suborders with known mating systems were calculated. This approach provides estimates of the total substitution rates leading from the outgroup species to the focal species and results in a sample size of 14 shorebird species with known mating systems because the outgroup species are effectively excluded. This approach neatly controls for the evolutionary time, as all pairwise comparisons between the outgroup and focal species reflect the same time period, but this requires phylogenetically controlled analyses owing to the inclusion of shared branches among focal species. In addition, the pairwise PAML model estimates are less affected by long and fast-evolving genes, as the *D*_*N*_/*D*_*S*_ estimates can be generated per orthologue and averaged without concatenation. Finally, the pairwise approach is not dependent on tree topology (and only assumes correct assignment of species to the Charadrii suborder).

To identify which of the 5239 orthologues were likely to be located on the Z chromosome in all species, only orthologues that were located on the Z chromosome in both the chicken and the common ringed plover *Charadrius hiaticula* were included in this group. Chicken gene locations could be taken directly from the chromosome-level genome assembly. The common ringed plover genome is less complete, and so placement of common ringed plover scaffolds on the Z chromosome was achieved by aligning 45X DNB-seq resequencing data from 10 individuals of known sex, 5 males and 5 females (sampling described in [63]). Sexes were confirmed in these 10 individuals through PCR amplification of the Chromo-Helicase-DNA binding gene using 2602F and 2669R primers [64]. Scaffolds covering >20 kb with approximately half as many aligned reads in females as males (a female-to-male ratio of 0.4–0.65) were assumed to represent parts of the Z chromosome. Approximately, 80% of genes located on the Z chromosome in one species were similarly located in the other, with the remaining 20% of genes excluded from the analysis. Out of the 5239 orthologues identified across all shorebirds, 106 were found to be located on the Z chromosome.

Molecular evolution is also affected by the variation in GC-biased gene conversion among different chromosome sizes [65]. To avoid the bias that would be introduced by the comparison of micro-chromosomes with the Z chromosome (a macro-chromosome), only autosomes 1–10 were included in the analysis (20–200 Mb), as these are of comparable size to the Z chromosome (83 Mb) [21]. The autosomal placement was taken from the chicken genome, and it was assumed to be largely conserved across Charadriiformes owing to the high level of synteny across birds in general [66]. Out of the 5239 orthologues, 3424 were located on autosomes 1–10. The remaining genes were excluded owing to their position on smaller autosomes, or because their position on the Z chromosome was inconsistent between the chicken and the common ringed plover.

### (d) Ancestral state reconstruction

For both the free-ratios PAML model and the pairwise PAML model, the differences between species in the pattern of substitutions reflect differences in molecular evolution occurring in the terminal branches. This means that any evolutionarily recent switches to a phenotype of role-reversed polyandry may result in weakened signatures of evolution under this mating system. We therefore wished to identify whether the sampled species with role-reversed polyandry had been evolving with this phenotype for a substantial proportion of their terminal branches. To investigate this, we performed an ancestral state reconstruction analysis.

All Charadriiformes with both mating system data and some genetic data for phylogenetic inference were included (215/330 charadriiform species). Mating system data were collated [44], and the phylogenetic tree was generated by downloading 10 000 likely trees with time-scaled branch lengths from birdtree.org [67]. These trees were then filtered to exclude any where the topology did not match our 34 charadriiform trees based on orthologous coding sequences. Only 2 trees out of 10 000 matched the topology based on orthologous coding sequences, and ancestral reconstruction analysis was performed using both of these. The low rate of topology matching was owing to our analyses’ placement of the grey plover *Pluvialis squatarola* outside of the *Charadriidae* clade (plovers, dotterels and lapwings) and the placement of the black skimmer *R. niger* outside the clade containing terns and gulls. Interestingly, in BirdTree.org trees, *R. niger* was placed with the gulls, which also does not match our own alternative phylogeny (where *R. niger* was placed with the terns). Recent attempts to resolve the charadriiform phylogeny have encountered similar difficulties regarding the placement of the *Rynchops* and *Pluvialis* genera [68]. Importantly, however, these discrepancies had no impact on the ancestral reconstruction owing to the lack of role-reversed polyandry within these clades. We performed ancestral rate reconstructions using the ‘ace’ command of the ‘ape’ R package [69]. Repeating this analysis with the second tree produced almost identical results. Note that non-shorebird charadriiforms were retained in the ancestral state reconstruction to improve basal node estimates.

### (e) Analysis of free-ratios PAML results

Following extraction of the free-ratios PAML model results using a custom R script, orthologues where the estimated synonymous (or non-synonymous) substitution rate exceeded 1 for any species were excluded, as estimates of the true number of substitutions become unreliable beyond 1 (a synonymous substitution rate of 1 already reflects all sites being substituted once, on average, since the most recent common ancestor). These steps led to a final dataset of 104 Z chromosome orthologues and 3354 orthologues from autosomes 1–10, each with estimated substitution rates for all 23 terminal shorebird branches. Substitutions on these orthologues were then summed (effectively concatenating the orthologues into a single sequence) and were divided by the number of sites available for each type of substitution (synonymous versus non-synonymous) in order to provide a single estimate of autosomal *D*_*N*_/*D*_*S*_ per terminal branch and a single estimate of Z chromosome’s *D*_*N*_/*D*_*S*_ per terminal branch.

To test for the presence of fast-Z in shorebirds, in general, a paired *t*‐test was used to evaluate whether the 23 concatenated Z chromosome’s *D*_*N*_/*D*_*S*_ estimates were higher than the 23 concatenated autosomal *D*_*N*_/*D*_*S*_ estimates. The 95% confidence intervals (CIs) for the autosomal and Z chromosome’s *D*_*N*_/*D*_*S*_ estimates were then generated for each terminal branch by bootstrapping to evaluate the reliability of the individual results. To test whether the strength of fast-Z was affected by role-reversed polyandry, Welch *t*-tests were used to test the impact of the mating system on the ratio of Z chromosome’s *D*_*N*_/*D*_*S*_ to autosomal *D*_*N*_/*D*_*S*_ (hereon, *D*_*NZ*_/*D*_*SZ*_:*D*_*NA*_/*D*_*SA*_, as shown by Wright *et al*. [6]). These *t*-tests specifically tested role-reversed polyandry (*n* = 4) versus all other known mating systems (*n* = 17) as well as role-reversed polyandry (*n* = 4) versus monogamy (*n* = 12). Additional Welch *t*-tests were used to investigate the impact of mating system on *D*_*NZ*_/*D*_*SZ*_ and *D*_*NA*_/*D*_*SA*_ independently.

### (f) Analysis of pairwise PAML results

The pairwise PAML analysis generated *D*_*N*_/*D*_*S*_ estimates for each outgroup species/focal species pair for each orthologue. As in the free-ratios model, orthologues, where the estimated synonymous or non-synonymous substitution rate exceeded 1 for any comparison, were excluded for the outgroup species in question. This not only ensured that unreliable estimates were excluded but also that orthologues were not excluded from all analyses owing to fast evolution within just one of the outgroup lineages. In addition, since orthologues were not concatenated for this analysis, only pairwise comparisons containing at least five synonymous substitutions and five non-synonymous substitutions for all focal species were retained. These steps led to a final dataset of 1826 orthologues from autosomes 1–10 and 64 orthologues from the Z chromosome (most excluded genes were removed on the basis of too few non-synonymous substitutions). For each outgroup species, the *D*_*N*_/*D*_*S*_ estimates for each autosomal orthologue were log(2)-transformed to normality, averaged across orthologues and then back-transformed to provide a single autosomal *D*_*N*_/*D*_*S*_ value per focal species. This process was repeated for the Z chromosome orthologues, and estimates of *D*_*NZ*_/*D*_*SZ*_:*D*_*NA*_/*D*_*SA*_ were then calculated. Finally, these values were averaged across all outgroups to provide a single estimate of *D*_*NA*_/*D*_*SA*_, *D*_*NZ*_/*D*_*SZ*_ and *D*_*NZ*_/*D*_*SZ*_:*D*_*NA*_/*D*_*SA*_ per focal species.

Phylogenetically controlled least squares (PGLS) models were used to test for an effect of the mating system on the strength of fast-Z (*D*_*NZ*_/*D*_*SZ*_:*D*_*NA*_/*D*_*SA*_) on the Z chromosome substitution ratio (*D*_*NZ*_/*D*_*SZ*_) and on the autosomal substitution ratio (*D*_*NA*_/*D*_*SA*_). All models were run using the ‘pgls’ function of the caper package [70]. The strength of the phylogenetic signal (Pagel’s λ) was estimated by maximum likelihood (ML), and model assumptions (normality of residuals and no heteroscedasticity) were checked visually through diagnostic plots. Initial analyses compared 4 focal shorebird species with role-reversed polyandry with 10 focal shorebird species with non-reversed mating systems. Separate models including only the monogamous comparison species (*n* = 6) were then constructed.

The pairwise PAML results were also used to investigate the relationship between the synonymous substitution rate and role-reversed polyandry. The orthologue selection criteria for this analysis were identical to that of the fast-Z analysis, with the exception that orthologues were not excluded on the basis of having too few non-synonymous substitutions. This resulted in synonymous substitution rates being based on 3191 autosomal genes and 101 Z chromosome genes. These values were then log(2)-transformed to produce a normal distribution and were averaged across outgroups following the same process as the *D*_*N*_/*D*_*S*_ analysis. The impact of role-reversed polyandry was analysed in three separate PGLS models—one model for autosomal orthologues (*D*_*SA*_), another for Z chromosome orthologues (*D*_*SZ*_) and a third investigating the Z synonymous substitution rate relative to the autosomal synonymous substitution rate (*D*_*SZ*_/*D*_*SA*_). These models also included generation time as a covariate using estimates from Bird *et al*. [71].

## 3. Results

### (a) Ancestral state reconstruction of role-reversed polyandry

**Figure 2.**
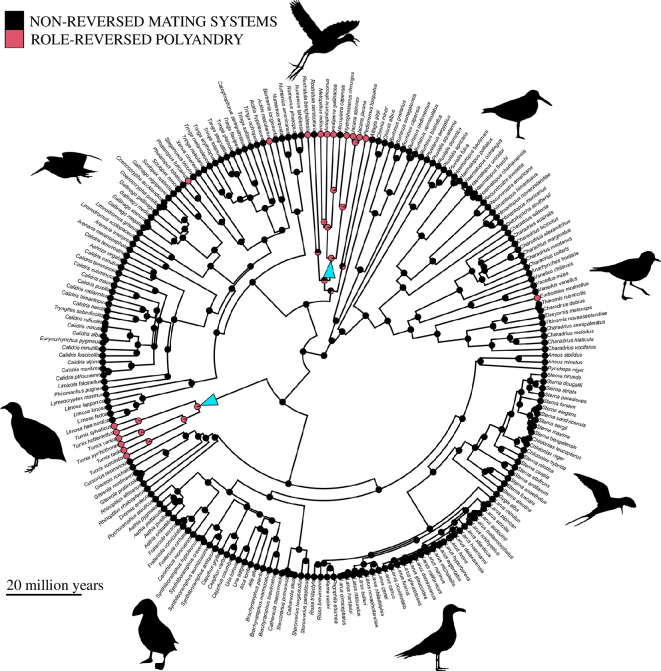
Ancestral reconstruction of role-reversed polyandry (pink) versus non-reversed mating systems (black) in Charadriiformes. Pie charts on nodes represent the likelihood that of the ancestor showing role-reversed polyandry versus non-reversed mating systems, and clades identified as evolving under role-reversed polyandry are highlighted with turquoise arrows (Jacanidae, top; Turnicidae, left). Note that we take a particularly strict definition of role-reversed polyandry, wherein >20% of females and <1% of males are polygamous within a mating season. The background phylogenetic tree is taken from BirdTree.org [67], with branches scaled by time.

Ancestral state reconstruction showed that at least half of our sampled role-reversed species have been evolving with this phenotype for a significant amount of time (figure 2). The *J. jacana* lineage is likely to have exhibited this phenotype for >38 million years, and the *T. velox* lineage is likely to have exhibited this phenotype for more than 31 million years. By contrast, the period with which the *Rostratula benghalensis* and *Pedionomus torquatus* lineages have been evolving with role-reversed polyandry is less certain owing to the few surviving members of these lineages and the mix of mating systems they exhibit. Predictions for the impact of role-reversed polyandry on molecular evolution should therefore be considered with more caution for these species. More generally, this analysis revealed that the strict form of role-reversed polyandry discussed here has evolved a minimum of five times within the Charadriiformes order.

### (b) Fast-Z in shorebirds

**Figure 3.**
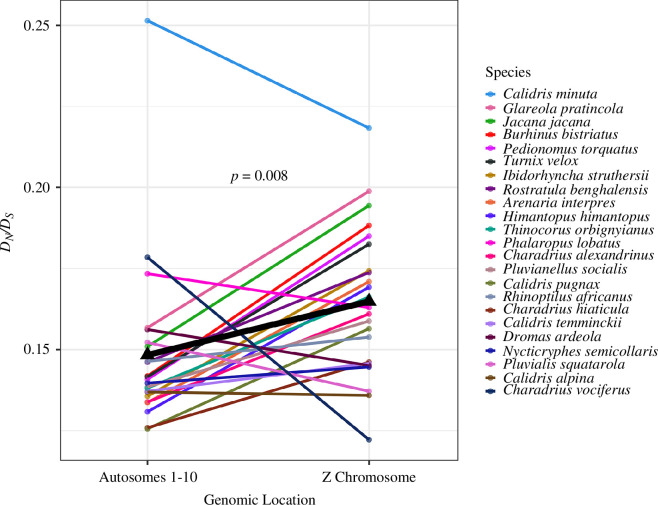
Comparison of *D*_*N*_/*D*_*S*_ estimates from concatenated orthologues located on autosomes 1–10 (3354 concatenated orthologues) versus those located in the Z chromosome (104 concatenated orthologues). Coloured lines and dots represent the results for each terminal branch (phylogeny in figure 1), while the black lines and triangles show the average values. Species are listed in the order of decreasing Z chromosome’s *D*_*N*_/*D*_*S*_ estimates. A paired *t*‐test found that Z chromosome *D*_*N*_/*D*_*S*_ estimates are significantly higher than autosomal *D*_*N*_/*D*_*S*_ estimates, consistent with a fast-Z effect in shorebirds (*p* = 0.008). Separate plots showing the bootstrapped CIs for each terminal branch can be found in electronic supplementary material, figure S1.

Using the terminal branch estimates of *D*_*N*_/*D*_*S*_ produced by concatenating 104 Z chromosome orthologues, and by concatenating 3354 orthologues from autosomes 1–10, a paired *t*‐test was used to test for the presence of fast-Z in shorebirds (data were paired by species). Consistent with a fast-Z effect operating in shorebirds, Z chromosome orthologues exhibited a significantly higher *D*_*N*_/*D*_*S*_ ratio than orthologues found on autosomes 1–10 (*t* = 2.90, *n* = 23, *p* = 0.008; figure 3). This effect remained significant when only considering macro-autosomes larger than the Z chromosome (autosomes 1–4, 91–200 Mb; *p* = 0.039), or when only considering macro-autosomes smaller than the Z chromosome (autosomes 5–10, 21–60 Mb; *p* = 0.001), confirming that the size of the Z chromosome (83 Mb) was not driving this effect. Note that Z chromosome *D*_*N*_/*D*_*S*_ estimates were based on relatively few substitutions for some terminal branches (varying from 360 substitutions in the smallest branch leading to *Calidris alpina* to 5740 substitutions in the longest branch leading to *T. velox*). Bootstrapping *D*_*N*_/*D*_*S*_ estimates by resampling orthologues revealed overlapping 95% CIs between Z chromosome’s *D*_*N*_/*D*_*S*_ and autosomal *D*_*N*_/*D*_*S*_ for all species except *T. velox* (electronic supplementary material, figure S1). Therefore, while there is good evidence that the Z chromosome tends to evolve faster than the autosomes in shorebirds, the assertion that fast-Z is occurring in each individual lineage should be made with caution.

### (c) Fast-Z and role-reversed polyandry

**Figure 4.**
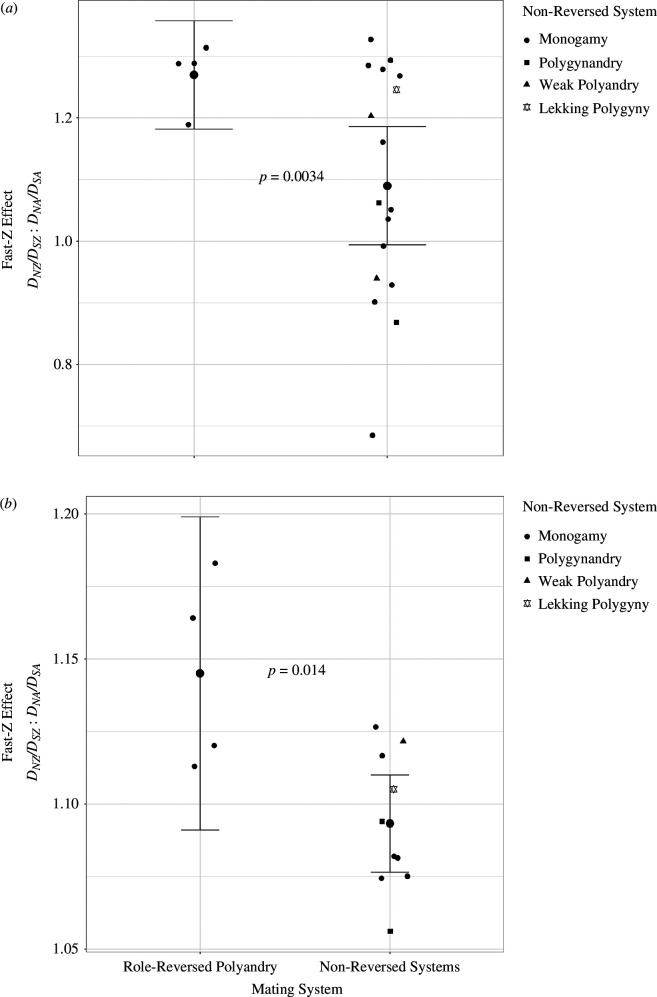
Fast-Z, as measured by *D*_*NZ*_/*D*_*SZ*_:*D*_*NA*_/*D*_*SA*_, is more severe under role-reversed polyandry than non-reversed mating systems. Smaller symbols represent data from each species, larger circles represent means and error bars represent 95% CIs. Non-reversed species are primarily monogamous, with exceptions denoted by symbols. (*a*) Faster fast-Z under role-reversed polyandry was found when calculating fast-Z based on evolution along the terminal branches of the shorebird phylogeny using a free-ratios PAML model. (*b*) The same result was found when calculating fast-Z based on the evolutionary change between the outgroup Charadrii shorebirds and the focal Scolopaci and Lari shorebirds using a pairwise PAML model.

The impact of the mating system on the ratio of Z chromosome *D*_*N*_/*D*_*s*_ to autosomal *D*_*N*_/*D*_*S*_ (*D*_*NZ*_/*D*_*SZ*_:*D*_*NA*_/*D*_*SA*_) was assessed using a Welch *t*‐test on the terminal branch substitution rates (concatenated over all orthologues). This test revealed significantly stronger fast-Z effects under role-reversed polyandry than non-reversed mating systems (figure 4*a*; table 1), which remained significant when only monogamous species were included in the control group (electronic supplementary material, table S1). Investigating the underlying causes of this, further Welch *t*-tests revealed that Z chromosome *D*_*N*_/*D*_*S*_ was higher under role-reversed polyandry, while there was no impact of the mating system on autosomal *D*_*N*_/*D*_*S*_ (table 1). Again, these results remained significant when only monogamous species were included in the control group (electronic supplementary material, table S1). These results are consistent with the expectation that different levels of selection on the Z chromosome drive differences in fast-Z strength between role-reversed polyandrous species and species with non-reversed mating systems (as opposed to differences in selection on the autosomes).

**Table 1.** Welch *t*‐test results testing the impact of role-reversed polyandry (*n* = 4 species) versus non-reversed mating systems (*n* = 17 species) on the strength of fast-Z (*D*_*NZ*_/*D*_*SZ*_:*D*_*NA*_/*D*_*SA*_), the Z chromosome evolutionary rate (*D*_*NZ*_/*D*_*SZ*_) and the autosomal evolutionary rate (*D*_*NA*_/*D*_*SA*_). Data based on free-ratios PAML models of terminal branches.

dependent variable	role-reversed species (95% CI)	non-reversed species (95% CI)	*t*–value	*p*‐value
*D*_*NZ*_/*D*_*SZ*_:*D*_*NA*_/*D*_*SA*_	1.27 (1.18–1.36)	1.09 (0.99–1.19)	3.4	0.0034
*D*_*NZ*_/*D*_*SZ*_	0.184 (0.170–0.197)	0.161 (0.148–0.173)	3.19	0.006
*D*_*NA*_/*D*_*SA*_	0.145 (0.137–0.152)	0.15 (0.135–0.166)	0.71	0.48

A second analysis focused on the pairwise comparisons of sequences between the Charadrii outgroup and the Scolopaci and Lari focal groups (see figure 1). Similar to the analysis of terminal branches, phylogenetically controlled models found that role-reversed polyandrous species showed a significantly stronger fast-Z effect than species with non-reversed mating systems (figure 4*b*; table 2), and this effect remained consistent when only monogamous species were included in the control group (electronic supplementary material, table S2). Also, largely in line with the results from the free-ratios PAML analysis, there was a trend towards faster evolution on the Z chromosome in role-reversed species, while no such trend was present on the autosomes (table 2). This trend remained when only monogamous species were included in the control group (electronic supplementary material, table S2).

**Table 2.** PGLS results testing the impact of role-reversed polyandry (*n* = 4 species) versus non-reversed mating systems (*n* = 10 species) on the strength of fast-Z (*D*_*NZ*_/*D*_*SZ*_:*D*_*NA*_/*D*_*SA*_), the Z chromosome evolutionary rate (*D*_*NZ*_/*D*_*SZ*_) and the autosomal evolutionary rate (*D*_*NA*_/*D*_*SA*_). Data based on pairwise PAML models.

dependent variable	role-reversed species (95% CI)	non-reversed species (95% CI)	Pagel’s lambda (ML)	*t*-value	*p*‐value
*D*_*NZ*_/*D*_*SZ*_:*D*_*NA*_/*D*_*SA*_	1.15 (1.109–1.20)	1.09 (1.08–1.11)	0^a^	2.89	0.014
D_NZ_/D_SZ_	0.120 (0.187–0.212)	0.192 (0.188–0.196)	1	1.84	0.091
*D*_*NA*_/*D*_*SA*_	0.175 (0.171–0.178)	0.176 (0.173–0.179)	1	0.8	0.44

^a^ Note that this model was unaffected by alterations in the assumed strength of the phylogenetic signal, remaining significant when *λ* = 1 (*p* = 0.036).

### (d) Synonymous substitution rates

Analysis focusing only on the synonymous substitution rates, again using a pairwise approach with the Charadrii shorebirds as an outgroup, found that role-reversed polyandry was associated with more frequent synonymous substitutions on both the Z chromosome and the autosomes (table 3). However, this result should be treated with caution, as the results were not replicated when only monogamous control species were included (electronic supplementary material, table S3). This inconsistency may reflect a confounding trend towards lower generation times in the role-reversed species, which was more severe when only monogamous control species were included (electronic supplementary material, tables S4; S5). There was no evidence that either mating system or generation time had any impact on the Z chromosome synonymous substitution rate *relative* to the autosomal synonymous substitution rate, suggesting that these effects are independent of fast-Z (table 3).

**Table 3.** PGLS results testing the impact of mating system (role-reversed polyandry (*n* = 4 species) versus non-reversed mating systems (*n* = 10 species) and generation time on: the Z chromosome synonymous substitution rate relative to the autosomal synonymous substitution rate (*D*_*SZ*_/*D*_*SA*_), the Z chromosome synonymous substitution rate log2(*D*_*SZ*_) and the autosomal synonymous substitution rate log2(*D*_*SA*_). Data based on pairwise PAML models.

dependent variable	model term	β (s.e.)^b^	Pagel’s lambda (ML^a^)	*t*-value	*p*‐value
*D*_*SZ*_/*D*_*SA*_	mating system	−0.0031 (0.018)	1	−0.18	0.86
	generation time	0.0005 (0.0033)	1	0.15	0.88
*D*_*SZ*_	mating system	0.26 (0.096)	1	2.69	0.021
	generation time	−0.025 (0.018)	1	−1.40	0.19
*D*_*SA*_	mating system	0.26 (0.11)	1	2.37	0.037
	generation time	−0.026 (0.021)	1	−1.25	0.24

^a^ Note that all model interpretations were unaffected by alterations in the assumed strength of phylogenetic signal.

^b^ β = regression coefficient. s.e. = standard error.

## 4. Discussion

A strong form of role-reversed polyandry has been present in two independent shorebird lineages for more than 30 million years (buttonquails and jacanas), and it has left a measurable signature of particularly fast evolution on the Z chromosome. This is consistent with sexual selection on polyandrous females driving particularly strong positive selection on partially recessive alleles on the Z chromosome [20,35]. If genetic drift and weak purifying selection were the primary driver of fast-Z variation, we would predict the opposite result of weaker fast-Z in polyandrous species owing to the higher variance in female reproductive success eroding the difference in the effective population size between the autosomes and the Z chromosome [22]. The signature of faster Z chromosome evolution under role-reversed polyandry was robust across two different models, with one model based on terminal branch *D*_*N*_/*D*_*S*_ estimates and another based on pairwise *D*_*N*_/*D*_*S*_ estimates using an outgroup. This consistency suggests a genuine effect; however, the results should nevertheless be treated with some caution owing to the small sample size of role-reversed species.

Generation times tended to be shorter in the sampled role-reversed polyandrous species. As a result, the relationship between role-reversed polyandry and the synonymous substitution rate could not be properly investigated. However, it is unlikely that this inequality in generation times confounded our wider analysis of fast-Z, as there was no impact of either generation time or role-reversed polyandry on the Z chromosome synonymous substitution rate relative to the autosomal synonymous substitution rate. This result also suggests that a more severe male mutation bias does not contribute to the effect of polyandry on fast-Z.

The elevated signature of fast-Z in a group of species with greater Z chromosome effective population sizes contrasts with most of the previous literature in birds, which has tended to implicate a central role of low effective population sizes and genetic drift in driving the fixation of harmful alleles on the Z chromosome [6,10,11,13,72]. Furthermore, the expectation of reduced female *N*_*e*_ is particularly well supported in polyandrous shorebirds, where even the census population size is male-biased [46,73]. However, this is the first analysis of fast-Z to include species with role-reversed polyandry, and the high variance in reproductive success for females under this mating system may lead to very strong sexual selection on females. Such strong selection on the polygamous females may increase the efficiency of positive selection acting on recessive alleles on the Z chromosome, which are exposed to selection in the hemizygotic females. This effect would increase the Z chromosome’s *D*_*N*_/*D*_*S*_ ratio and may be overwhelming the impact of the reduced Z chromosome’s genetic drift. If reproductive success reflects the overall fitness as suggested by ‘good genes’ theories of sexual selection, such enhanced positive selection under polygamy need not be limited to genes specifically associated with reproduction [35]. This interpretation is in line with recent research suggesting that the increase in sexual selection associated with polygamy can purge harmful alleles from a bird population despite the overall loss of effective population size generally associated with higher variance in reproductive success [36].

One alternative mechanism that may explain the accelerated rate of Z chromosome evolution in role-reversed polyandrous shorebirds is sexually antagonistic selection, which occurs when the optimal allele for a locus differs between males and females [74]. Sexually antagonistic selection is expected to be more common among polygynous and polyandrous species owing to the increase in sexual dimorphism relative to monogamy [75,76]. There is also evidence that sexually antagonistic alleles occur at a higher frequency on the Z chromosome in birds, as the Z chromosome contains a higher frequency of alleles harmful to females than the autosomes, and genes in older regions of the Z chromosome more commonly exhibit sex-biased expression than those regions which are more recent in this chromosome [6,72,77,78]. Therefore, if sexually antagonistic genes evolve at a faster rate than other genes (e.g. owing to sex-limited expression leading to greater genetic drift [79,80]), the greater prevalence of sexually antagonistic genes on the Z chromosome may contribute to the strength of fast-Z, and the greater frequency of sexual antagonism under greater sexual dimorphism may contribute to the increase in fast-Z strength under role-reversed polyandry. A role of sexually antagonistic selection in driving fast-Z is also consistent with much of the previous research in birds, including the excess of both non-synonymous polymorphisms and non-synonymous substitutions on the Z chromosome [10,11,13], the acceleration of fast-Z in polygynous Galliformes [6], the excess of Z chromosome genetic diversity among more sexually dimorphic bird species [81] and a (non-significant) trend towards faster fast-Z among more sexually dimorphic bird species [72].

Further research is therefore required to understand why role-reversed polyandrous shorebirds show stronger fast-Z effects, and the ideal test would be an analysis of polymorphisms. A central role of sexually antagonistic selection would predict an excess of Z-chromosomal non-synonymous polymorphisms under role-reversed polyandry owing to the maintenance of sexually antagonistic loci in species with greater sexual dimorphism. By contrast, enhanced sexual selection on polyandrous females would predict a reduction in the Z chromosome’s non-synonymous polymorphisms, as partially recessive harmful alleles are more effectively purged.

## 5. Conclusion

Here, we used published shorebird genomes to analyse the effect of role-reversed polyandry on fast-Z evolution. Species with role-reversed polyandry showed a stronger fast-Z signature than the control species, which were predominantly monogamous. This may reflect greater levels of sexual selection on recessive beneficial alleles in hemizygotic females increasing fast-Z in polyandrous shorebirds. However, we highlight that the role of sexual antagonism may be underappreciated and may also lead to particularly fast Z chromosome evolution in sexually dimorphic species.

## Data Availability

The genome assemblies used were all previously published [36,54] and can be found deposited at NCBI SRA (accession PRJNA545868 and PRJNA739535), with some also found at CNGBdb (accession CNP0001928). The R code, as well as orthologue alignments, datasets and phylogenies, have been deposited onto Zenodo [82]. Supplementary material is available online [83].
